# A novel method to find out sensory neuron tracts in the Drosophila brain

**DOI:** 10.1186/1471-2202-16-S1-P35

**Published:** 2015-12-18

**Authors:** Chao-Chun Chuang

**Affiliations:** 1 National Center for High-Performance Computing, Taiwan, Republic of China

## 

How receptions of sensory inputs information turn into perceptions in our brain? To address these questions, we proposed method reconstructs the neuronal tracts by applying the shortest path graph algorithm between functional regions in the Drosophila brain. With these neuronal tracts, we analyze and draw a network diagram of projection neurons (PNs) relaying sensory input to higher brain centers in the Drosophila brain. Drosophila is a widely used genetic model system for understanding human biology [1,2]. While distinctively different in gross anatomy, insect brains and mammalian brains are both made of neural circuits with a cohort of similar gene expression governing the basic demands of life. With this network diagram, numerous unexpected local networks and inter-regional pathways were found from our initial analysis of sensory systems including olfactory, gustatory, auditory, and vision circuits [3].

**Figure 1:**
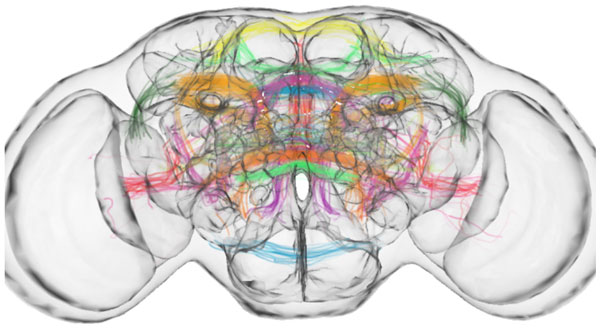
**Numerous PNs tracts relaying sensory inputs - including olfactory, visual, auditory, and gustatory - to higher brain centers were discovered**.

## Conclusion

The network diagram shows hierarchical structure, small-world characteristics, and is composed of functional modules corresponding to the sensory modalities. This ultimate goal of such an atlas is to identify connectivity between neurons for understanding the function/circuit relationships.

## References

[B1] Li CY Chuang CC Hua TT Chen CC Dickson BJ Greenspan RJ A comprehensive wiring diagram of the protocerebral bridge for visual information processing in the Drosophila brain Cell Reports 2013 3 5 1739 1753 23707064 10.1016/j.celrep.2013.04.022

[B2] Lee PC Chuang CC Chiang AS Ching YT High-throughput computer method for 3d neuronal structure reconstruction from the image stack of the Drosophila brain and its applications PLoS Computational Biology 2012 8 9 e1002658 23028271 10.1371/journal.pcbi.1002658 PMC3441491

[B3] Chiang AS Lin CY Chuang CC Chang HM Hsieh CH Yeh CW Three-dimensional reconstruction of brain-wide wiring networks in Drosophila at single-cell resolution Current Biology 2011 21 1 1 11 21129968 10.1016/j.cub.2010.11.056

